# *ag85A* loss promotes free mycolate accumulation and sensitizes plasma membrane domain to disruption

**DOI:** 10.1128/jb.00240-26

**Published:** 2026-07-14

**Authors:** Takehiro Kado, Shivangi, Paige H. Bosworth, Jake Jordan, Joel S. Freundlich, M. Sloan Siegrist, Yasu S. Morita

**Affiliations:** 1 Department of Biology, Missouri State University 735791, Springfield, Missouri, USA; 2 Department of Pharmacology, Physiology, and Neuroscience, New Jersey Medical School, Rutgers University 67206, Newark, New Jersey, USA; 3 Department of Microbiology, University of Massachusetts 196202, Amherst, Massachusetts, USA; 4 Molecular and Cellular Biology Graduate Program, University of Massachusetts Amherst 14707, Amherst, Massachusetts, USA; University of Notre Dame, Notre Dame, Indiana, USA

**Keywords:** antigen 85A, mycolic acids, membrane domain, membrane permeability, mycobacteria

## Abstract

The mycomembrane of mycobacteria, which is composed primarily of long-chain mycolic acids, is critical for cell survival, structural integrity, and resistance to environmental stress; however, its underlying synthesis mechanisms remain incompletely understood. This study investigated the role of the mycolyltransferase Ag85A, a key enzyme in mycomembrane synthesis, in regulating plasma membrane domains and cell envelope organization in *Mycobacterium smegmatis*. Using ∆*ag85A* deletion mutants, we combined microscopy, subcellular fractionation, mass spectrometry, and lipid analysis to evaluate changes in membrane structure, intracellular chemical accumulation, and lipid composition. The deletion of *ag85A* led to destabilization of the plasma membrane domain, increased chemical accumulation, and altered cell envelope lipid compositions. Consistent with prior studies, Δ*ag85A* accumulated mycolates, specifically in the free lipid pool rather than in the covalently linked cell wall fractions. In addition, the accumulation of an uncharacterized glycolipid species was absent or reduced in the wild-type and complemented strains; these findings indicate that Ag85A loss alters the distributions of mycolic acid-containing lipids and is associated with destabilization of the plasma membrane domain, highlighting a functional link between the processes of lipids and plasma membrane architecture.

**IMPORTANCE:**

The unique cell envelope of mycobacteria plays a vital role in survival by protecting against immune responses and antibiotic treatments. In this study, we examined the consequences of deleting *ag85A*, a well-characterized mycolyltransferase that contributes to the assembly of the mycomembrane lipid layer. In addition to known phenotypes, such as increased drug sensitivity and disrupted biofilm formation, our data reveal alterations in membrane-associated lipid distributions and suggest a possible link between membrane synthesis and plasma membrane organization. These findings provide new insight into the broader cellular impact of Ag85A loss and suggest avenues for further investigation into how lipid biosynthesis interfaces with membrane architecture. Understanding this relationship may inform new therapeutic strategies that destabilize the protective envelope of pathogenic mycobacteria.

## INTRODUCTION

The outer membrane in diderm bacteria provides structural integrity and stability to the bacterial shape (1) while also acting as a barrier against antibiotics and other external stressors (2). In mycobacteria, the outer membrane—called the mycomembrane—is rich in long-chain fatty acids known as mycolic acids (3). The mycomembrane is a defining and highly specialized outer layer of mycobacteria that has long been recognized as a key antibiotic target (3, 4). Clinically used, narrow-spectrum antibiotics exploit this envelope architecture through distinct mechanisms, including ethambutol, which inhibits arabinogalactan synthesis (5–7), and isoniazid, which targets enzymes in the mycolic acid biosynthetic pathway (8).

Two key mycolic acid-containing molecules, namely, trehalose mono-mycolate (TMM) and trehalose di-mycolate (TDM), play essential roles in bacterial survival and pathogenicity (3, 9–14). In addition to ethambutol and isoniazid, compounds such as SQ109, delamanid, and pretomanid have been reported to alter mycolate profiles, although disruption of TMM/TDM synthesis does not appear to be their primary mode of action (15–19). Central to TMM/TDM synthesis are enzymes from the antigen 85 family, specifically Ag85A, -B, and -C. These enzymes catalyze the synthesis of TMM and TDM, can regenerate TMM from TDM, and can also attach mycolates to arabinogalactan (20, 21). Their abundant presence and critical function in mycolate metabolism make them attractive targets for understanding the role of the mycomembrane in mycobacterial survival strategies. Among the Ag85A, Ag85B, and Ag85C isoforms, Ag85A has received the most attention because of its strong transferase activity (22).

Research involving the knockout of *ag85A* in *Mycobacterium smegmatis*, *Mycobacterium bovis*, and *Mycobacterium tuberculosis* has revealed several phenotypic changes. Antibiotic sensitivity and accumulation are increased in *M. smegmatis* and *M. tuberculosis* (23–25). The growth rates of macrophages are reduced in *M. bovis* and *M. tuberculosis* (26, 27)*,* and biofilm formation is disrupted in *M. smegmatis* and *M. tuberculosis* (24, 28, 29). Additionally, transposon screening revealed that *ag85A* deletion may delocalize the plasma membrane domains (30), known as the inner membrane domain (IMD) (30–32), where cell envelope precursors are synthesized in *M. smegmatis* (31, 33). These findings suggest that in addition to its established role in mycomembrane biosynthesis, Ag85A may also contribute to the organization of plasma membrane domains in association with changes in antibiotic susceptibility, intracellular growth, and biofilm development.

In this study, we investigated how the disruption of mycomembrane biosynthesis through deletion of the mycolyltransferase *ag85A* influences plasma membrane organization in *M. smegmatis*. Our analyses revealed the accumulation of free mycolates and destabilization of plasma membrane domains under stress in the *ag85A* deletion mutant. These results indicate that changes in the composition of the mycomembrane can influence the spatial organization of plasma membrane domains. This study explores the connection between cell envelope biosynthesis and membrane organization, offering new directions for understanding mycobacterial cell envelope biology and identifying potential vulnerabilities for therapeutic intervention.

## RESULTS

### Accumulation of the membrane fluidizer in ∆*ag85A*

**Fig 1:**
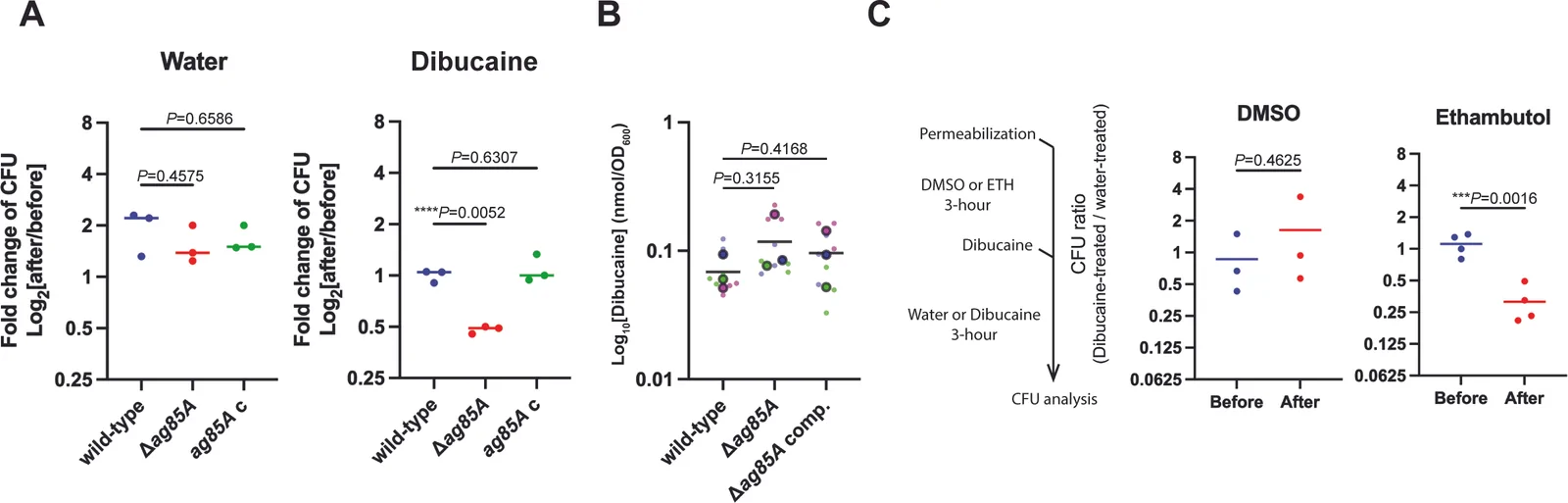
Ag85A protected *M. smegmatis* from membrane fluidizer accumulation. (**A**) Dot plots showing the log₂ fold changes in CFUs measured before and after 3 h of treatment. The left panel represents water-treated samples, and the right panel represents dibucaine-treated samples. Statistical significance was assessed using the Kruskal–Wallis test, followed by Dunn’s multiple comparisons test, with *P* values indicated in the graph. (**B**) Intracellular accumulation of dibucaine was quantified by liquid chromatography‒mass spectrometry and visualized by using GraphPad Prism. Small circles represent individual technical replicate values within each biological replicate (distinguished by color), large circles indicate the mean of each biological replicate, and bars indicate the overall mean of biological replicates. (**C**) Cells were pretreated with either ethambutol or DMSO (vehicle control) for 3 h. After preincubation, the cells were washed and then treated with dibucaine or water for an additional 3 h. CFU counts were obtained for each group both before and after dibucaine treatment, and CFU ratios were calculated by dividing the CFU of the dibucaine-treated group by the CFU of the water-treated group. *P* values were determined by paired t tests.

In previous transposon sequencing experiments, cells were treated with the membrane fluidizer dibucaine, a compound known to transiently disrupt plasma membrane domains (34, 35). After 3 h of treatment, the cells were washed and incubated overnight (30). This approach was designed to identify mutations that confer increased sensitivity to membrane domain disruption and reduced fitness during recovery. In this screen, *ag85A* resulted in fewer transposon insertions under dibucaine treatment (30), suggesting that deletion of *ag85A* may heighten the susceptibility to dibucaine by destabilizing the membrane structure or slowing membrane domain reorganization during recovery. To determine whether ∆*ag85A* is sensitive to dibucaine or is simply delayed in recovery, the deletion mutant was treated with dibucaine for 3 h, and the fold changes in the numbers of colony-forming units (CFUs) were assessed after treatment. In a quantitative assay in which the CFU fold changes before and after 3 h of treatment were measured, all strains exhibited modest 1.2–2-fold increases following mock treatment (Fig. 1A, left and Table S1). In contrast, dibucaine treatment resulted in no net change in CFU levels for the wild-type and complemented strains, reflecting the bacteriostatic effect of dibucaine (30). The ∆*ag85A* mutant, however, showed a twofold reduction in CFUs, indicating that approximately half of the cells lost viability during dibucaine exposure (Fig. 1A, right and Table S1). Our previous work using other deletion mutants identified by Tn-seq under membrane fluidizer treatment revealed delayed growth kinetics without a reduction in CFUs following exposure (30, 34), suggesting that those deletions primarily affected recovery dynamics. In contrast, the reduced number of CFUs observed for ∆*ag85A* suggests that the recovery of the mutant is not merely delayed but is more sensitive to dibucaine.

While *ag85A* deletion increases dibucaine sensitivity, the underlying cause of cell death in the mutant is still not defined. Previous studies have shown that the loss of *ag85A* increases mycomembrane permeability (25), which may increase dibucaine accumulation in cells. To test whether this increased sensitivity reflects enhanced drug penetration, we quantified dibucaine accumulations in cells. We quantified dibucaine in the cell using liquid chromatography–mass spectrometry (LC‒MS) (36, 37). wild-type and ∆*ag85A* strains were treated with dibucaine (200 μg/mL) for 3 h, and the dibucaine taken up by the cells was subsequently extracted with a mixture of methanol, acetonitrile, and water (MAW) (2:2:1, vol/vol/vol). LC‒MS analysis revealed no statistically significant differences among the strains (Fig. 1B); however, a trend toward increased dibucaine accumulation in the ∆*ag85A* mutant was observed compared with that in the wild-type strain (Fig. 1B). To rule out the possibility that strain-specific background effects on dibucaine ionization might influence dibucaine measurements, we spiked dibucaine into lysates from the wild-type, ∆*ag85A*, and complemented strains that were treated with water only. We then compared the dose–response curves of dibucaine in different lysates with those of dibucaine in MAW (2:2:1, vol/vol/vol). The dose response curves of dibucaine were similar across all the samples (Fig. S1), indicating that the observed trends in dibucaine accumulations were not due to matrix effects.

The slightly increased accumulation of dibucaine in the Δ*ag85A* strain led us to hypothesize that the combined effect of a weakened cell envelope and the accumulation of dibucaine culminates in cell death. To assess whether disruption of the cell envelope integrity increases the susceptibility of *M. smegmatis* to dibucaine, we treated wild-type *M. smegmatis* with ethambutol, a known inhibitor of arabinogalactan biosynthesis (3, 25, 38–42), followed by dibucaine treatment. Susceptibility was assessed by calculating the ratio of CFUs in the dibucaine-treated group to those in the water-treated control group. A CFU ratio less than 1 indicated that ethambutol treatment reduced cell viability in the presence of dibucaine. Treatment with DMSO (a vehicle control for ethambutol) did not significantly differ in terms of the number of CFUs between the dibucaine-treated group and the water-treated group, suggesting that no sensitization occurred under these conditions. In contrast, pretreatment with a sublethal concentration of ethambutol resulted in a significant decrease in CFUs upon dibucaine exposure, resulting in a CFU ratio less than 1 (Fig. 1C; Table S2). These results indicate that ethambutol-mediated mycomembrane permeabilization sensitized *M. smegmatis* to dibucaine, enhancing its antibacterial effect. Taken together, these data suggest that the defective mycomembrane in the ∆*ag85A* mutant*,* combined with dibucaine exposure, leads to enhanced cell death. These results validated our previous Tn-seq analysis, which revealed *ag85A* as an important gene for resistance to dibucaine treatment.

### Delocalized membrane domain under membrane fluidizer treatment in Δ*ag85A*

**Fig 2:**
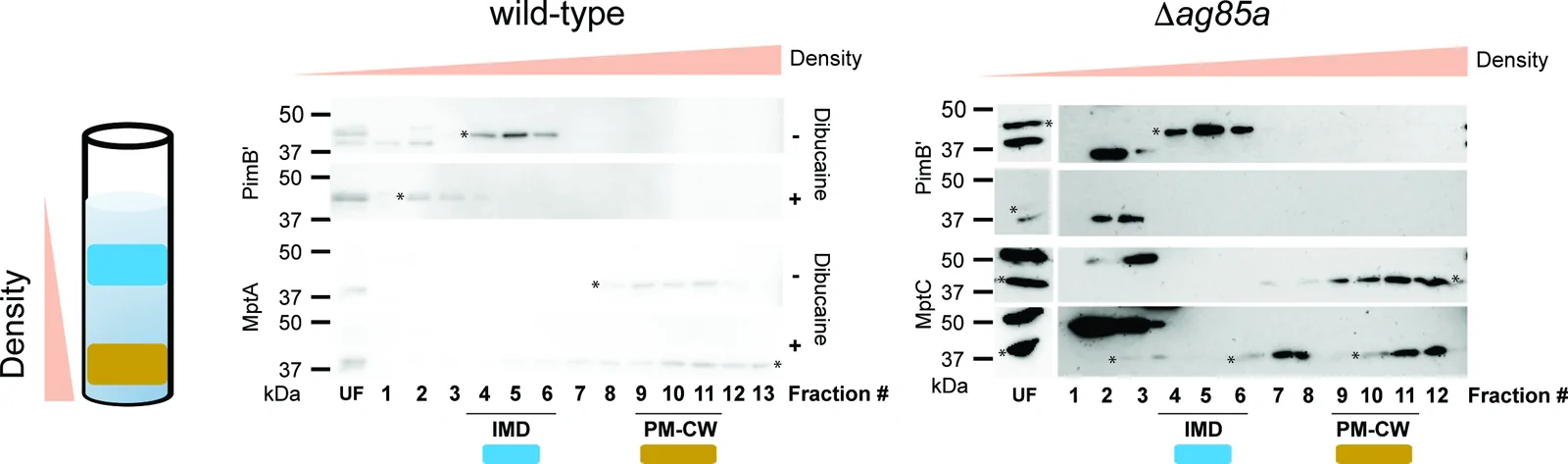
Dibucaine treatment shifted the PM-CW to lighter fractions in Δ*ag85A*. (Left) Diagram of IMD and PM-CW fractionation after ultracentrifugation. (Right) To visualize IMD and PM-CW, PimB′, MptA, and MptC, which are established marker proteins (31), were analyzed by western blotting. Images are representative of two independent experiments. Asterisks indicate the proteins analyzed. UF, unfractionated.

Next, we examined the membrane domain organization of wild-type and ∆*ag85A* in response to dibucaine. Cells were lysed by nitrogen cavitation, and the resulting samples were separated by sucrose gradient fractionation. IMD and plasma membrane-cell wall (PM-CW) domains were present in fractions 4–6 and 8–12 (Fig. 2,), which is consistent with previous literature (31, 33, 34, 43). The microscopy image of the IMD marker protein also revealed standard subpolar localization in the absence of dibucaine (Fig. S2). Upon dibucaine treatment, the IMD marker protein PimB′ was not detected in Δ*ag85A*, with only a faint signal detected in the unfractionated sample (Fig. 2), which may represent an effect of dibucaine on the expression or stability of PimB′. The PM-CW domain appeared in fragmented distributions across fractions 2–3, 6–7, and 10–12 in Δ*ag85A* (Fig. 2, asterisks). In wild-type cells, marker proteins of the IMD and PM-CW domains were detected broadly across the gradient after dibucaine treatment, as described in a previous study (30). This distribution pattern in the wild type was interpreted as domain plasticity, in which membrane domains undergo reversible reorganization without complete loss of domain identity (30). In contrast, the discontinuous PM-CW distribution that was observed in Δ*ag85A* suggested a more severe disruption of domain organization. These findings suggest that the lateral separation of the plasma membrane in the ∆*ag85A* mutant is more susceptible to disruption by membrane fluidizers than it is in the wild type.

### Accumulation of free mycolates in Δ*ag85A*

Ag85A enzymatically mediates the synthesis of TDM from TMM or the reverse reaction (21); therefore, deletion of this gene may affect the composition of the mycomembrane. Disrupted structural integrity could be a leading factor in dibucaine sensitivity, as well as the unique patterns of membrane domain disruption observed after dibucaine treatment. However, the changes in the composition of the mycomembrane resulting from *ag85A* deletion are not fully understood. We further characterized the mycomembrane biochemically to investigate how the deletion of *ag85A* affects the structure of the mycomembrane.

Deletion of *ag85A* has been reported to alter TDM levels, although the direction of this change differs between the two studies. Nguyen et al. reported a significant decrease in TDM in a Δ*ag85A* mutant on the basis of thin-layer chromatography and quantitative analysis of ^14^C-acetate-labeled lipids, revealing an approximately 45% reduction in TDM compared with that in wild-type cells (24). In contrast, Touchette et al. analyzed total cellular mycolates and reported greater accumulations of TMM and TDM in the deletion mutant than in the wild type and higher TDM synthesis when TMM with an N-alkyne moiety was used for click chemistry (44).

**Fig 3:**
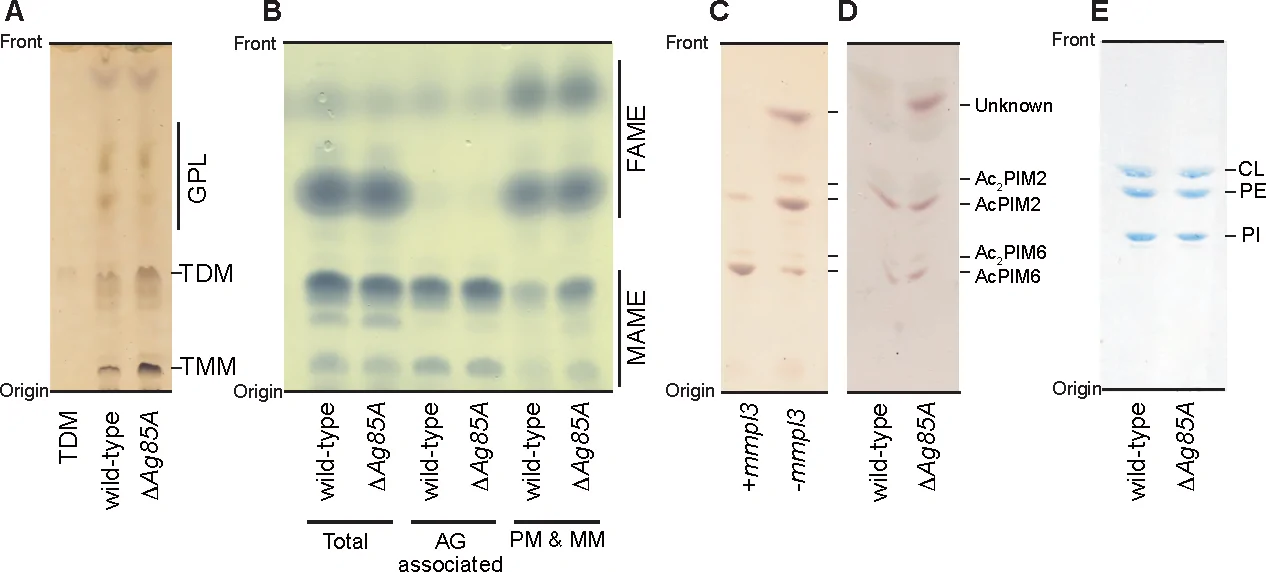
Mycolates and an unknown glycolipid were accumulated in ∆*ag85A*. (**A**) TMM and TDM accumulated in the ∆*ag85A* mutant. TMM and TDM were resuspended in chloroform/methanol/water (9:1:0.1, vol/vol/vol), chromatographed using chloroform, and visualized by orcinol. (**B**) Mycolic acids and fatty acids were methylated and chromatographed as methyl esters using a solvent containing petroleum ether and acetone (95:5, vol/vol). (**C and D**) PIMs from the indicated mutants were separated using chloroform/methanol/13 M ammonia/1 M ammonium acetate/water (180:140:9:9:23, vol/vol/vol/vol/vol) as the mobile phase and visualized by orcinol. The *mmpl3*-depleted strain was used as a control, in which a mycomembrane precursor (TMM) accumulates in the plasma membrane. (**E**) Phospholipids were chromatographed using the same solvent as in panels C and D and visualized by molybdenum blue. CL, cardiolipin; PE, phosphatidylethanolamine; PI, phosphatidylinositol; and PIM, phosphatidylinositol mannoside. All experiments were repeated at least twice. Bands were assigned as the expected molecules on the basis of migration patterns reported in previous literature.

To quantify total TMM and TDM in cells, we used the method of Touchette et al. to extract total membrane lipids from mycobacterial cells and confirmed the TMM and TDM accumulations in the deletion strain (Fig. 3A). To assess the distribution of mycolates between free lipids and cell wall-associated forms, we first extracted free TMM and TDM, after which the delipidated cells were subjected to alkaline hydrolysis to obtain arabinogalactan-bound mycolates (45), which were subsequently converted to mycolic acid methyl esters (MAMEs) for analysis (Fig. 3B). The results suggested that compared with the wild-type and complement strains, the free lipid fraction of Δ*ag85A* accumulated (Fig. 3B; Fig. S3A).

Next, we wanted to determine whether TMM accumulates in the mycomembrane or plasma membrane of Δ*ag85A*. TMM is an intermediate that is produced in the inner leaflet of the plasma membrane and is transported to the periplasmic leaflet by the MmpL3 flippase (46). Previous studies using chemical inhibition or depletion of MmpL3 have shown that disruption of TMM transport leads to the accumulation of TMM in the plasma membrane, which is accompanied by alterations in plasma membrane properties (47–50). Since phosphatidylinositol mannosides (PIMs) remodel their fatty acids in response to plasma membrane perturbations, including membrane-fluidizing conditions (35), we reasoned that PIM fatty acid remodeling may be observed when *mmpL3* expressions are disrupted by CRISPRi. TMM accumulation resulting from *mmpl3* knockdown (47, 50) caused the accumulation of diacylated species of PIMs (Fig. 3C). In contrast, the PIM profiles were largely unaffected in Δ*ag85A* (Fig. 3D), and there were no other changes in the levels of major phospholipid species, including cardiolipin, phosphatidylethanolamine, and phosphatidylinositol (Fig. 3E).

Together, these data indicate that although TMM accumulation occurs in the Δ*ag85A* mutant, it does not elicit the same plasma membrane-associated lipid remodeling that is observed upon MmpL3 depletion. These findings suggest that TMM accumulations in Δ*ag85A* are insufficient to perturb the plasma membrane and elicit PIM remodeling, or that TMM accumulates in the mycomembrane instead.

### Accumulations of iodine-reactive and alkaline-sensitive glycolipids in Δ*ag85A*

**Fig 4:**
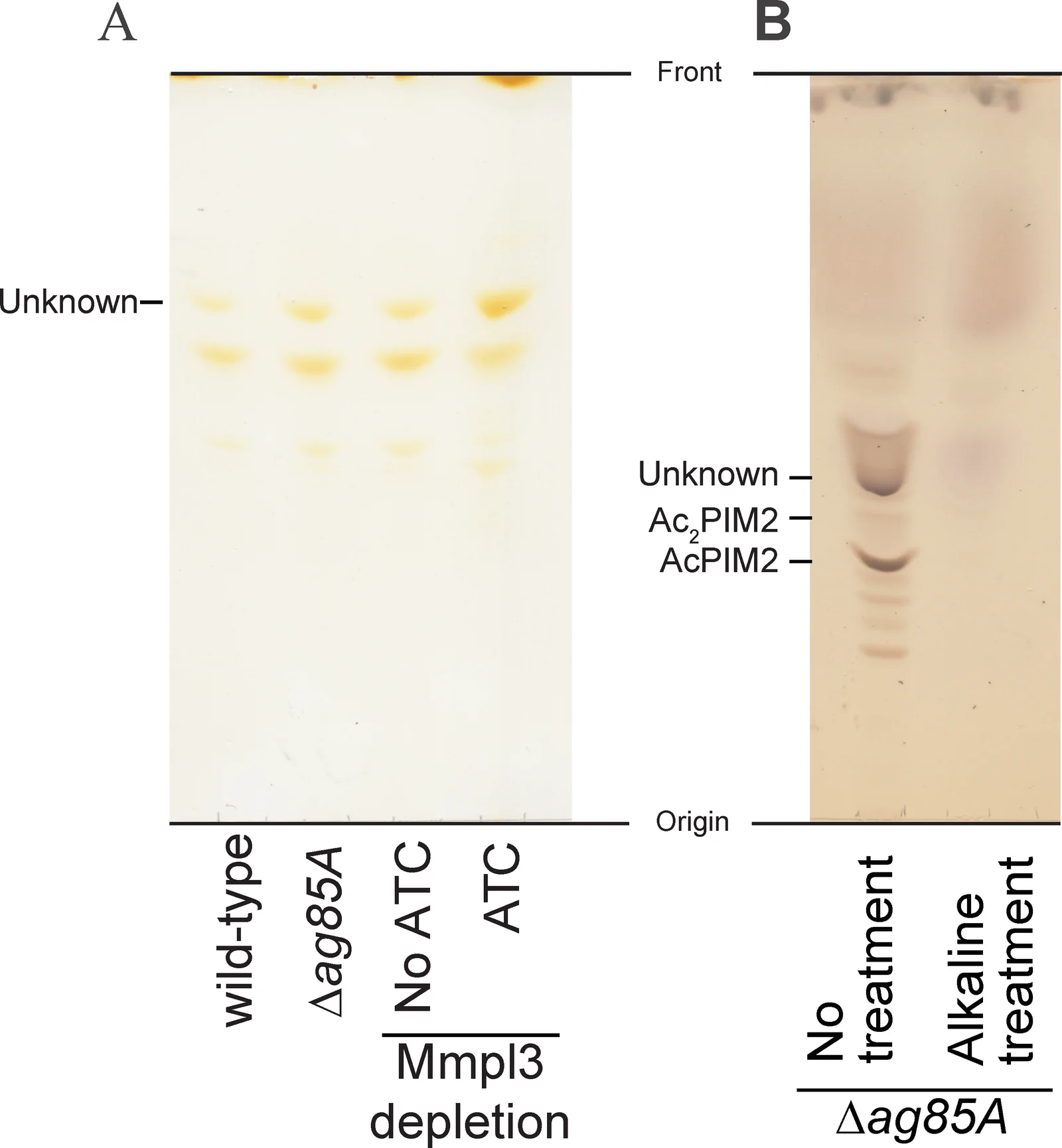
Unknown glycolipids were stained by iodine and susceptible to alkaline hydrolysis. (**A**) The unknown glycolipid was chromatographed using the same solvent as in Fig. 3C and D and visualized by iodine vapor. (**B**) Lipid extracts were treated with 0.2 M NaOH and resuspended in chloroform, methanol, and water (10:10:3, vol/vol/vol). The samples were chromatographed using the same solvent as in panel A and visualized by orcinol.

In addition to TDM accumulations, thin-layer chromatography (TLC) analysis revealed a unique band with a retention factor of 0.65–0.70 in the *ag85A* deletion and *mmpl3* depletion strains, which was absent or faint in the wild-type and complemented strains (Fig. 3D; Fig. S3B). This band stained positively with orcinol reagent, which specifically detects carbohydrates, indicating the production of a glycolipid. Glycolipids with high hydrophobicity are typically glycopeptidolipids (GPLs) or lipooligosaccharides (LOSs), both of which are components of the mycomembrane. Although LOS production has been reported to be absent in the *Mycobacterium smegmatis* mc^2^155 strain (51), the genes required for LOS biosynthesis are conserved, and their expressions may have been induced by the deletion of *ag85A*. To provide structural insights into unknown lipids, we performed iodine staining and alkaline hydrolysis. We found that this lipid is detectable by iodine (Fig. 4A) and is sensitive to alkaline hydrolysis (Fig. 4B). Iodine is a weak electrophile that reversibly binds to C=C double bonds, suggesting that this lipid may contain unsaturated fatty acids. Alkaline sensitivity suggests the presence of acyl-linked fatty acids. Since GPLs are alkaline resistant, these lipids may not be GPLs. While these findings are consistent with the possibility that the unknown lipid is LOS, further matrix-assisted laser desorption/ionization time-of-flight (MALDI-TOF) analysis did not reveal an increase in LOS signals (Fig. S4), which appeared as peaks at *m*/*z* 1,689 and 1,717 in LOS-producing *M. smegmatis* in a previous study (52). At present, we are unable to determine the structure and precise localization of this lipid. Nonetheless, its accumulation highlights an alteration in envelope lipid homeostasis that accompanies *ag85A* deletion. Definitive identification and functional characterization of this lipid will be needed to determine its role in determining its structure or permeability.

## DISCUSSION

Ag85A is present across mycobacteria and plays a critical role in the synthesis of the mycobacterial cell envelope (24, 53). As a member of the Ag85 complex, it catalyzes the transfer of mycolic acids to arabinogalactan and TMM, contributing to the formation and maintenance of the mycomembrane, a key determinant of envelope integrity and pathogenicity (11–14, 54–56). The plasma membrane of mycobacteria is organized into specialized membrane domains that coordinate the biosynthesis of various envelope components, including peptidoglycan, arabinogalactan, and mycolic acid-containing lipids (31, 33, 57, 58). Previous work has demonstrated that changes in the composition of the cell wall or plasma membrane lipids can influence the architecture of these domains (33, 34, 59). For example, deletion of the peptidoglycan synthase PonA2 delays *de novo* formation of plasma membrane domains (34). Loss of the cyclopropane fatty acid synthase Cfa, which converts oleic acid to tuberculostearic acid in plasma membrane phospholipids, also disrupts plasma membrane domain formation (30). Previous studies have demonstrated that the absence of *ag85A* leads to increased sensitivity to and accumulation of certain drugs (24, 25, 60) and changes in the composition of the mycomembrane (44). In this study, we investigated the effects of *ag85A* deletion using intrabacterial drug accumulation assays, membrane domain fractionation, fluorescence microscopy, and targeted lipid analysis. Our findings indicate that the absence of *ag85A* weakens the membrane domain structure and is associated with the accumulation of free mycolates.

Several mechanistic possibilities could explain how *ag85A* loss resulted in the fragmented delocalization of plasma membrane domains. One explanation is that TMM becomes stalled within the lipid transport machinery. The flippase MmpL3 is proposed to move TMM across the plasma membrane (48, 61), but additional components are likely involved in translocating mycolates to the mycomembrane. Recent studies have identified candidate proteins involved in this process, including TMM transport factor A (TtfA) (62), a predicted lipid transport protein (LprG) (44), and a lipoprotein (LpqN) (63). We hypothesize that *ag85A* deletion disrupts this lipid trafficking process, creating a bottleneck that leads to TMM accumulation at the membrane interface. This buildup may act as a physiological signal that feeds back to the plasma membrane, altering its spatial organization and ultimately destabilizing the membrane domain that coordinates envelope biogenesis.

A second possibility is that TMM accumulates in the outer leaflet of the plasma membrane. While MmpL3 depletion likely causes TMM buildup in the inner leaflet (48, 62) and the accumulation of TMM-induced PIM acylation (Fig. 3D), we did not observe PIM acylation in the Δ*ag85A* strain. However, the relationship between outer leaflet accumulation of the TMM and PIM modification remains uncharacterized; thus, this possibility cannot be excluded. Given that TMM consists of unusually long and rigid carbon chains (3), enrichment of TMM in the outer leaflet of the plasma membrane could alter membrane fluidity. Since previous studies have shown that membrane fluidity influences the organization of plasma membrane domains in bacteria (30, 33, 34, 64), biophysical changes could interfere with domain integrity even in the absence of obvious PIM remodeling, highlighting that TMM accumulation in the outer leaflet is a plausible contributor to the dibucaine-sensitive plasma membrane domain.

A third possibility is that Ag85A contributes more directly to the cross-talk between the mycomembrane and plasma membrane. Both mycobacteria and *Escherichia coli* are diderm bacteria, and studies in *E. coli* have shown that outer membrane disruption destabilizes plasma membrane integrity (1) and even bacterial morphology (65). In the case of Δ*ag85A*, defective maturation of the mycomembrane may weaken the signaling or physical coupling normally required to stabilize plasma membrane domains. Rather than being driven solely by TMM buildup, the phenotype could therefore arise from broader changes in outer membrane composition and structure that fail to support proper domain organization, propagating stress back to the plasma membrane.

Together, these scenarios highlight how *ag85A* loss disturbs mycolate homeostasis, whether by stalling the TMM transport machinery, driving its enrichment in the plasma membrane outer leaflet, or altering mycomembrane maturation. In each case, the imbalance could destabilize plasma membrane domains, linking outer-layer lipid defects with domain disorganization. This perspective strengthens the emerging view that plasma membrane domains and cell envelope structures are interdependent and dynamically coupled, with feedback from cell envelope integrity shaping membrane domain organization and stability.

The feedback between the cell envelope and plasma membrane domains is supported by prior genetic studies (59). Disruption of the expressions of envelope synthesis enzymes, such as *ponA2*, a peptidoglycan synthase (34), or *cfa*, a fatty acid-methylating enzyme (30), leads to defects in plasma membrane domain formation. These findings suggest that the structural integrity of the envelope is not only a downstream output of domain function but also a critical upstream regulator of domain architecture. Our data add to this model by implicating Ag85A, a key player in mycolate transfer. We propose that the loss of *ag85A* impairs mycomembrane maturation, which in turn disrupts the structural integrity of plasma membrane domains. Together, these data support the view that plasma membrane domains and the cell envelope exist in a self-reinforcing loop, wherein defects in lipid transport or envelope structure propagate back to the membrane level to coordinate or constrain biosynthetic activities.

While we favor the interpretation that defective mycomembrane maturation perturbs plasma membrane domain organization through feedback, an alternative explanation is that this phenotype arises from generalized stress that is imposed by envelope defects. A recent manuscript revealed that strains with highly permeabilized mycomembranes displayed increased antibiotic sensitivity, along with elevated TMM accumulations (66). Compared with those of the wild type, a weakened mycomembrane exposes cells to greater environmental stress, and prior studies have shown that stress conditions, such as nutrient starvation or a prolonged stationary phase, can drive IMD delocalization (32). In our experiments, the Δ*ag85A* strains showed trends toward higher intracellular dibucaine accumulations, although the effect was not statistically significant. This outcome may indicate that the loss of *ag85A* reduces cellular tolerance to stress and that the degree of IMD delocalization reflects stress-induced reorganization rather than a direct consequence of a mycolate imbalance. Importantly, recent work has demonstrated that IMD delocalization is controlled by the proton gradient (67), suggesting that these shifts are not purely passive outcomes of envelope instability but can also represent an active, regulated response to stress.

Several additional mechanisms may contribute to or result from the disrupted lipid homeostasis observed in the Δ*ag85A* mutant, offering insight into both the origin and broader impact of mycolate accumulation. The first hypothesis focuses on the production of an orcinol-positive glycolipid that emerged specifically in the *ag85A* mutant and *mmpl3* depletion strains. This glycolipid may represent a compensatory adaptation. Both the accumulation and the mislocalization of TMM and TDM could trigger the synthesis of unknown glycolipids, potentially as a compensatory response to restore cell envelope homeostasis. Second, it remains possible that other Ag85 isoforms, such as *Ag85B* or *Ag85C*, are upregulated in the absence of *ag85A*, leading to excess TMM synthesis beyond the capacity of the transport and incorporation machinery. This enzymatic compensation could further contribute to lipid imbalance and domain instability.

Maintaining a balanced mycolate composition in the outer cell layer is critical for envelope impermeability and membrane organization. Loss of *ag85A* disturbed this balance, leading to lipid buildup and changes in membrane organization. A precise molecular connection between excess mycolate and domain disruption remains undefined, but findings point toward a shared system that links lipid biosynthesis with membrane integrity. Genes, such as *ag85A*, *ponA2*, and *cfa*, help shape membrane domains (30, 34). When any of these proteins are removed, cells suffer from a disorganized membrane domain, resulting in systemic envelope stress, growth defects, and drug sensitivity. Given the presence of the membrane domain in *M. tuberculosis* (68), these findings position plasma membrane domains as promising targets for future drug development.

## MATERIALS AND METHODS

### Bacterial strains and growth conditions

The *M. smegmatis* mc^2^-155 strain was used as the wild-type strain. The Δ*ag85A* mutant and the complemented strain were obtained from Dr. Anil Ojha (Wadsworth Center, NY). The depletion strain of *mmpl3* was generated by introducing a CRISPRi (*mmpl3*: gatataatctgggaGACAGACTGGCTGCCCTCGTCgtttttgtactcga) construct provided by MSRdb (69). The depletion construct was introduced into the wild type by electroporation (70). The cells were grown at 37°C in Middlebrook 7H9 broth supplemented with 11 mM glucose, 14.5 mM NaCl, and 0.05% (vol/vol) Tween 80. To deplete *mmpl3*, anhydrotetracycline (final concentration: 50 ng/mL; Cayman Chemical, Michigan, USA) was added, and the culture was incubated overnight.

### Colony-forming units

CFUs were determined as previously described (30). In brief, the wild-type, ∆*ag85A*, and complemented strains were grown to the stationary phase, then diluted and grown to the log phase. The cultures were treated with water (vehicle control) or 200 µg/mL dibucaine (Millipore Sigma, Massachusetts, USA) at 37°C for 3 h, after which the cultures were serially diluted in 7H9 media and plated. Colonies were counted after approximately 48 h of incubation at 37°C following plating on Middlebrook 7H10 agar.

### CFU assay for ethambutol pretreatment

Cells were pretreated with either ethambutol (50 μg/mL) or DMSO (vehicle control) for 3 h. After incubation, the cells were washed to remove any residual ethambutol or DMSO. The cells were then treated with dibucaine or water for an additional 3 h. CFU counts were obtained for each group both before and after dibucaine treatment. To compare the effects of the treatments, the CFU ratios were calculated by dividing the CFU counts of the dibucaine-treated group by the CFU counts of the water-treated group. These ratios were determined both before and after treatment.

### Intrabacterial drug accumulation measurement

*M. smegmatis* (wild-type, Δ*ag85a*, or the complement strain) cultures were grown in 7H9 broth supplemented with 11 mM glucose, 14.5 mM NaCl, and 0.05% (vol/vol) Tween 80 until they reached the mid-log phase (OD_600_ ~0.6). The cultures were then divided into three tubes for technical triplicate measurements. Water (vehicle control) or dibucaine was added, and OD_600_ readings were taken at 0 h, followed by incubation at 37°C, after which the samples were collected at 1 h. After incubation, the OD_600_ values were measured, and 3× 1 mL samples were collected and pelleted by centrifugation at 13,000 rpm (14,549 rcf) for 10 min. The pellets were resuspended in 1 mL of 0.85% NaCl and pelleted again, after which the supernatant was removed. For cell lysis, the pellets were resuspended in 1 mL of MAW solution (2:2:1, vol/vol/vol) and stored at −80°C. Bead beating (6.5 m/s for 30 s, 6 cycles, with ice cooling for 2 min between cycles) with Lysing Matrix B (MP Biomedical, California, USA) was performed to lyse cells (FastPrep24 5G, MP Biomedicals, OH), followed by centrifugation. The supernatants were transferred to 0.22-µm spin-X centrifuge tube filters (Corning, New York, USA) and spun at 13,000 rpm (14,549 rcf) for 10–15 min at 4°C. The samples were stored at −80°C until LC‒MS analysis.

### LC‒MS analysis of intrabacterial-accumulated drugs/compounds

The samples were analyzed using a Poroshell 120 EC-C18 column (2.1 × 50 mm; 1.9 µm particle size) and an Agilent 1290 Infinity II HPLC system connected to an Agilent InfinityLab LC/MSD system. A standard gradient program (shown as the percentage of acetonitrile) was used to construct a solvent system with water/acetonitrile that was acidified with 0.1% formic acid: 0 to 0.4 min, 5%; 0.4 to 0.5 min, 5%–30%; 0.5 to 3.0 min, 30%–95%; 3.0 to 3.3 min, 95%; and 3.3 to 3.6 min, 95%–5%. We utilized a flow rate of 0.5 mL/min and a post-run time period of 1 min. The construction of the calibration curves was performed as detailed previously (37).

### Membrane fractionation

The membrane was fractionated as previously described (43). In brief, 5 mL of seed culture was inoculated into three 2-L glass flasks, each containing 500 mL of supplemented 7H9 medium, and incubated at 37°C for 16–18 h. At mid-log phase (OD₆₀₀ = 0.5–1.0), the culture was transferred to 1,000-mL bottles and centrifuged at 8,000 rpm for 15 min at 4°C using an F9S-4×1000Y rotor (Sorvall). The resulting pellet was resuspended in 50 mM HEPES/NaOH buffer (pH 7.4) and transferred to preweighed 50-mL conical tubes. The samples were then centrifuged at 4,000 rpm for 10 min at 4°C using an Eppendorf A-4-81 rotor. After centrifugation, the supernatant was removed, and the pellet was resuspended in 40 mL of 50 mM HEPES/NaOH buffer (pH 7.4). The samples were subsequently centrifuged again under the same conditions, after which the final pellet was weighed.

The pellet was then resuspended at a ratio of 1 g per 4 mL in lysis buffer (25 mM HEPES/NaOH [pH 7.4], 20% sucrose, and 2 mM EGTA) containing protease inhibitor mix (Roche). Cell disruption was carried out by nitrogen cavitation at 1,850–2,200 psi for 30 min, with pressure released every 10 min, and this process was repeated for a total of three cycles. A sucrose gradient (20%–50% wt/vol) was prepared using a BioComp Gradient Master. Following cavitation, the lysate was centrifuged at 4,000 rpm for 10 min at 4°C, and the supernatant was carefully transferred to a new tube, avoiding the top layer. This centrifugation and transfer step was repeated once. A 1.2-mL volume of clarified lysate was carefully layered onto the sucrose gradient and centrifuged at 35,000 rpm (218,000 × *g*) for 6 h at 4°C using a Beckman L8-70M ultracentrifuge equipped with an SW40 swing bucket rotor. Gradient fractions were collected using a BioComp gradient collector, and 450 µL from each fraction was aliquoted into separate tubes and stored at –80°C.

### SDS-PAGE and western blotting

SDS-PAGE and western blotting were performed as described previously (30). For sample preparation, equal volumes of each fraction were loaded to assess the protein distributions across the gradient rather than absolute protein abundances. Specifically, 12 µL of each fraction was mixed with 4 µL of 4× sample loading buffer and incubated on ice for 30 min. A protein ladder was prepared by mixing 10 µL of Precision Plus Protein Kaleidoscope Standards (Bio-Rad, California, USA) in an Eppendorf tube and incubating at 95°C for 5 min. The samples and ladder were then loaded onto SDS-PAGE gels and run at a constant current of 25 mA per gel.

Following electrophoresis, the proteins were transferred onto membranes at 14 V overnight using a Bio-Rad PowerPac 1000 system (Bio-Rad, California, USA). The membranes were blocked in 5% skim milk prepared in PBS containing 0.05% Tween 20 (PBS-T) for 1 h at room temperature. Blots were incubated overnight at 4°C with rabbit polyclonal anti-PimB′, anti-MptA, or anti-MptC antibodies (71). The membranes were then incubated with a horseradish peroxidase-conjugated donkey anti-rabbit antibody (Cytiva) at a 1:4,000 dilution. The protein bands were visualized using a homemade enhanced chemiluminescence reagent, and images were captured using an Amersham ImageQuant 800 system (Cytiva).

### Microscopy and image analysis

Microscopy and image analysis were performed as previously described (34). In brief, a 5-µL aliquot of bacterial culture was placed on an agar pad (1% agarose in water) positioned on a glass slide. Images were captured using a Nikon Eclipse E600 microscope (Nikon Eclipse Ti with ×100 objectives, N.A. = 1.30) or Leica CTR6500 microscope (Leica HCX PL APO with ×100 objectives, N.A. = 1.40). Cell outlines were traced using Oufti software (72). MATLAB scripts were used to generate intensity profiles (57).

### Lipid extraction and analysis

Analysis of MAME was performed as described previously (45). Briefly, 50 mg of mycobacterial cells was mixed with 2 mL of 15% tetrabutyl ammonium hydroxide and heated overnight at 100°C. The next morning, the mixture was diluted with 2 mL of water, 1 mL of dichloromethane, and 250 μL of iodomethane and then stirred for 30 min. The upper aqueous layer was discarded, and the lower organic layer was washed with 3 mL of 1 M HCl, followed by 3 mL of water. If needed, the volume was reduced. The residue was dried under a stream of nitrogen gas, dissolved in dichloromethane, transferred to a polypropylene tube, and evaporated under nitrogen. The dried MAMEs were dissolved in 0.2 mL of toluene and 0.3 mL of acetonitrile and incubated at 4°C for 1 h, after which any insoluble debris was discarded to yield the MAME extract. For TLC, the MAMEs are spotted on aluminum-backed silica sheets, developed in petroleum ether/acetone (95:5, vol/vol), and visualized by spraying with 5% ethanolic phosphomolybdic acid, followed by charring at 110°C. Arabinogalactan-associated mycolic acid was separated from mycolic acids associated with free lipids by first removing free lipids in chloroform/methanol (2:1, vol/vol).

Analysis of phospholipids and glycolipids was performed as described previously (35, 43, 73). In brief, 50 OD_600_ units of cells were harvested into a preweighed 50-mL conical tube and centrifuged at 4,000 rpm (3,220 × *g*) for 10 min. The supernatant was discarded, and the process was repeated until all the cells were collected into a single tube. The residual supernatant was removed with an aspirator, and the tube was weighed to calculate the pellet weight. The pellet was extracted with 20 volumes of chloroform/methanol (2:1) for 1.5 h at room temperature, ensuring complete suspension. After centrifugation, the supernatant was transferred to a fresh 15-mL conical tube. A second extraction was performed with 10 volumes of chloroform/methanol (2:1), followed by centrifugation and transfer of the supernatant. A third extraction was then carried out using chloroform/methanol/water (1:2:0.8), and the supernatants were combined.

From the combined extract, 800 µL was dried under nitrogen, resuspended in 100 µL of water, and mixed with 200 µL of water-saturated butanol. After vortexing and centrifugation for 30 s, the upper phase was transferred to a new tube. This butanol extraction was repeated, and 100 µL of water was added to the combined butanol phase for a final round. The upper phase was collected, dried in a speed-vac, and resuspended in 20 µL of water-saturated butanol.

For TLC, the tank was equilibrated with chloroform/methanol/13 M NH_3_/1 M NH_4_Ac/water (180:140:9:9:23). Three microliters of the sample was spotted on an HPTLC plate (Millipore Sigma, California, USA) and developed for approximately 45 min. The plates were dried and stained with one of the following: orcinol reagent at 100°C for 5 min, cupric acetate at 150°C for 10–20 min, iodine vapor at room temperature for 10–20 min, or molybdenum blue reagent at room temperature for 10 min.

### Alkaline hydrolysis of the lipid extract

The desired volume of lipid-containing solution was dried under a nitrogen stream and resuspended in 100 µL of 0.2 M NaOH in methanol or, for the methanol-only control, in 100 µL of methanol. The samples were incubated at 40°C for 2 h. The reaction was neutralized with 1.2 mL of glacial acetic acid and then dried under nitrogen. The dried sample was resuspended in 100 µL of water, mixed with 200 µL of water-saturated butanol, vortexed, and centrifuged for 30 s, after which the upper phase was transferred to a new tube. A second extraction was performed with an additional 100 µL of water-saturated butanol. The combined butanol phases were mixed with 100 µL of water and extracted again, and the final butanol phase was dried using a speed-vac. The dried material was resuspended in 10 µL of chloroform/methanol/water (10:10:3, vol/vol/vol), and the tube was subsequently washed with 5 µL of chloroform/methanol (2:1, vol/vol). The sample was then spotted onto a TLC silica gel 60 sheet (Merck). Plates were predeveloped with 100% chloroform, dried, and developed with chloroform/methanol (9:1, vol/vol). After drying, the plates were sprayed with orcinol reagent, baked at 100°C for 5 min, and imaged using a computer scanner.

### MALDI-TOF-MS analysis of the lipid extracts

Lipid extracts were analyzed by matrix-assisted laser desorption/ionization time-of-flight mass spectrometry (MALDI-TOF-MS). The 1-butanol extract was mixed in a 1:1 volume ratio with a saturated solution of 2,5-dihydroxybenzoic acid prepared in 70% acetonitrile with 0.1% trifluoroacetic acid. The samples were spotted onto a stainless steel MALDI target plate and allowed to air-dry at room temperature. Mass spectra were acquired using a Bruker ultrafleXtreme MALDI-TOF/TOF mass spectrometer (Bruker Daltonics, Billerica, MA, USA) operated in linear positive-ion mode with an accelerating voltage of 20,000 V. Spectra were collected by averaging 500 laser shots per sample. External calibration was performed using a peptide or lipid standard mixture to ensure mass accuracy.

## SUPPLEMENTAL MATERIAL

Supplemental tables and figures jb.00240-26-s0001.pdf Tables S1 and S2, and Figures S1 to S4.

ASM does not own the copyrights to Supplemental Material that may be linked to, or accessed through, an article. The authors have granted ASM a non-exclusive, world-wide license to publish the Supplemental Material files. Please contact the corresponding author directly for reuse.
