# Supplementary material for: ag85A loss promotes free mycolate accumulation and sensitizes plasma membrane domain to disruption
Source: J Bacteriol. 2026 Jul 14;208(8):e00240-26. doi: 10.1128/jb.00240-26 (PMC13531430; doi:10.1128/jb.00240-26)
Supplement: Supplementary file 1 [file jb.00240-26-s0001.pdf]

Supplementary Table 1. Viability of wild-type, Δ*ag85A*, and complemented strains before and after dibucaine treatment.

**Water**	**Replicate**	**Before**	**After**	**Fold change**
wild-type	1	3.40E+06	7.80E+06	2.29
	2	4.80E+06	1.06E+07	2.21
	3	3.80E+06	5.00E+06	1.32
Δ*ag85A*	1	9.20E+06	1.14E+07	1.24
	2	8.40E+06	1.68E+07	2.00
	3	2.60E+06	3.60E+06	1.38
ag85A comp.	1	4.40E+06	6.60E+06	1.50
	2	6.60E+06	9.80E+06	1.48
	3	1.20E+06	2.40E+06	2.00
**Dibucaine**	**Replicate**	**Before**	**After**	**Fold change**
wild-type	1	3.80E+06	4.00E+06	1.05
	2	4.40E+06	4.60E+06	1.05
	3	4.20E+06	3.80E+06	0.90
Δ*ag85A*	1	1.06E+07	5.20E+06	0.49
	2	1.32E+07	6.00E+06	0.45
	3	2.80E+06	1.40E+06	0.50
ag85A comp.	1	5.40E+06	5.40E+06	1.00
	2	1.04E+07	9.80E+06	0.94
	3	1.20E+06	1.60E+06	1.33

CFUs were measured before and after treatment with water (control) or dibucaine. Values represent three independent biological replicates. Fold change was calculated as CFU after treatment divided by CFU before treatment.

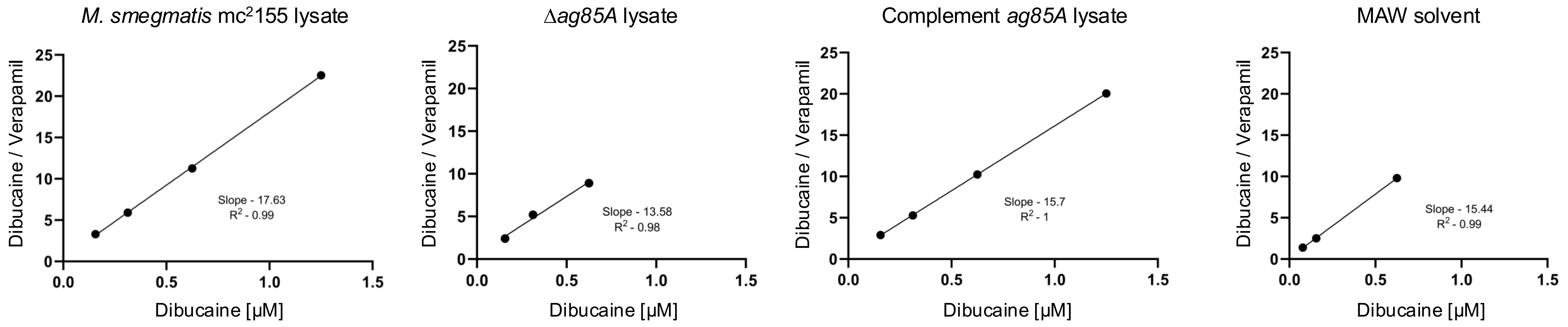

	M. smegmatis mc²155 lysate (S_B)	Δ*ag85A* lysate (S_B)	Complement *ag85A* lysate (S_B)	MAW solvent (S_M)
Dibucaine sensitivity	17.6	13.6	15.7	15.4
Matrix effect (S_B/S_M)	1.14	0.88	1.02	

Note: S_B stands for sensitivity of dibucaine in biological lysates of different strains, and S_M stands for sensitivity in MAW solvent.

Supplementary figure 1: There is no significant matrix effect observed among different strains. Calibration curves of dibucaine in the presence of different cell lysates and MAW were made by plotting the dibucaine concentrations vs. ratio of dibucaine to verapamil. The slope in the presence of cell lysate was compared to slope in the presence of MAW.

Supplementary table 2. Effect of pre-permeabilization on cell survival in response to dibucaine.

After permeabilization								
	Permeabilization	DMSO			Ethambutol			
	Replicate	1	2	3	1	2	3	4
CFU at 0 hours	A: For water treatment	2.40E+07	5.60E+07	4.00E+06	3.20E+07	3.20E+07	1.94E+07	2.98E+07
	B: For dibucaine treatment	1.60E+07	2.40E+07	6.00E+06	3.20E+07	4.40E+07	2.50E+07	2.38E+07
Ratio X (B/A)	Before dibucaine	0.6667	0.4286	1.5000	1.0000	1.3750	1.2887	0.7987

After water or dibucaine treatment								
	Permeabilization	DMSO			Ethambutol			
	Replicate	1	2	3	1	2	3	4
CFU at 3 hours	a: Water treated	1.76E+08	9.60E+07	4.80E+06	8.00E+06	3.80E+07	1.46E+06	1.72E+06
	b: Dibucaine treated	1.00E+08	9.00E+07	1.62E+07	2.60E+06	8.80E+06	7.20E+05	3.60E+05
Ratio Y (b/a)	After dibucaine	0.5682	0.9375	3.3750	0.3250	0.2316	0.4932	0.2093

CFUs were measured under permeabilization conditions (DMSO or ethambutol) followed by water or dibucaine exposure. A and B represent CFU before treatment for water and dibucaine conditions, respectively, and the ratio X (B/A) indicates relative starting cell abundance. After treatment, a and b represent CFU following water or dibucaine exposure, respectively, and the ratio Y (b/a) indicates relative survival after dibucaine treatment. Values represent independent replicates.

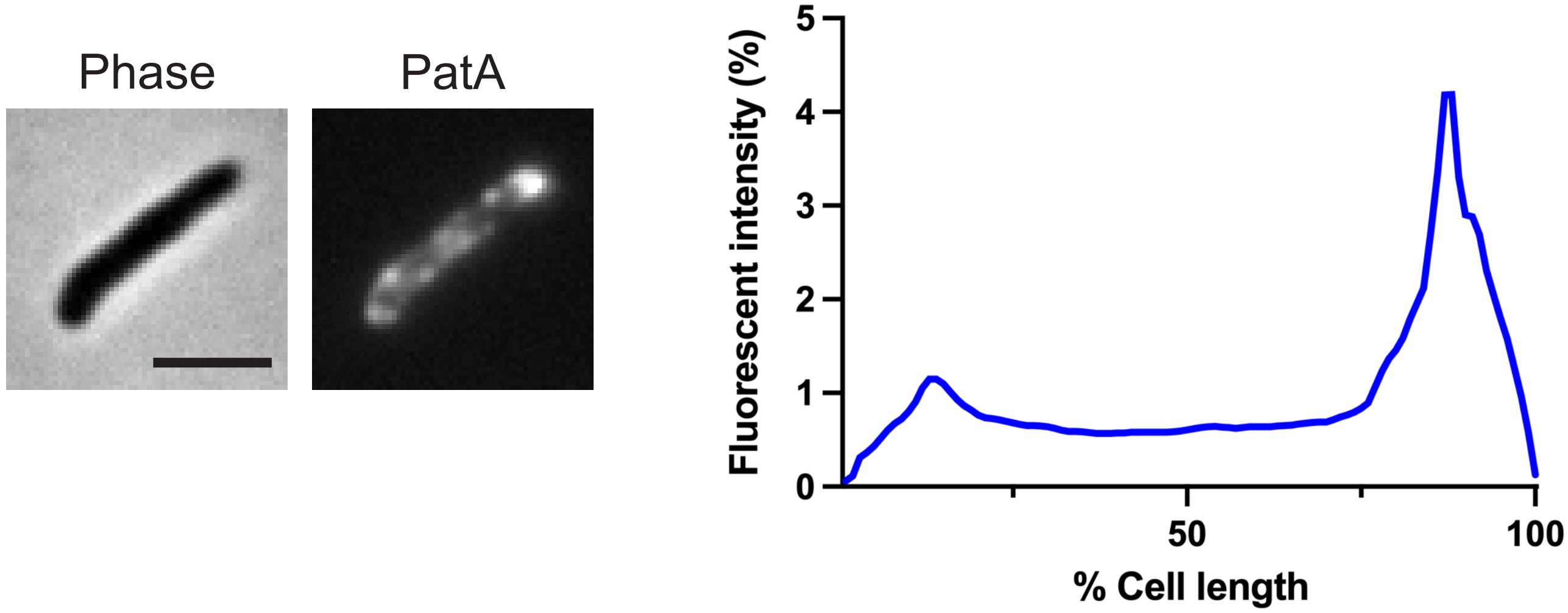

Supplementary Figure 2. The deletion of *ag85A* did not induce IMD delocalization. Localization of PatA, an IMD marker protein, was imaged during log-phase growth. Images are representative of three independent biological experiments. Fluorescence distribution profiles of the PatA-Dendra2 fusion protein were quantified from three independent biological replicates. Lines represent the average signal from all analyzed cells (n = 50). Fluorescence intensity was normalized to cell length and total cellular fluorescence. Scale bar, 2.5 μm.

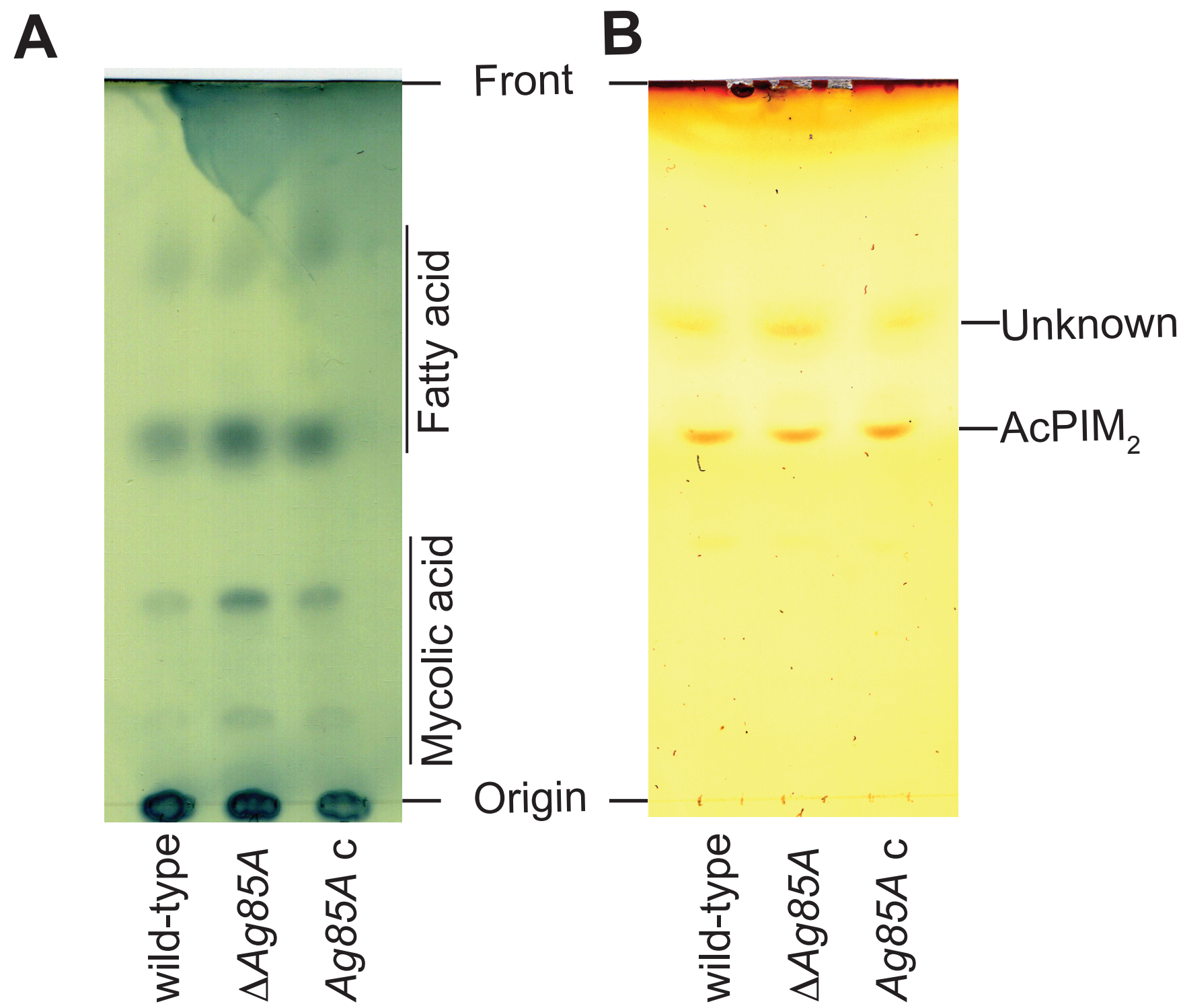

Supplementary Figure 3. (A) Mycolic acids and fatty acids from free lipid fractions (panel A) were methylated and separated using TLC with a petroleum ether:acetone (95:5, v/v) solvent system, followed by staining with 5% phosphomolybdic acid. (B) TLC plate visualized with orcinol after separation using a solvent system of chloroform, methanol, 13 M ammonia, 1 M ammonium acetate, and water (180:140:9:9:23, v/v/v/v/v), followed by orcinol staining.

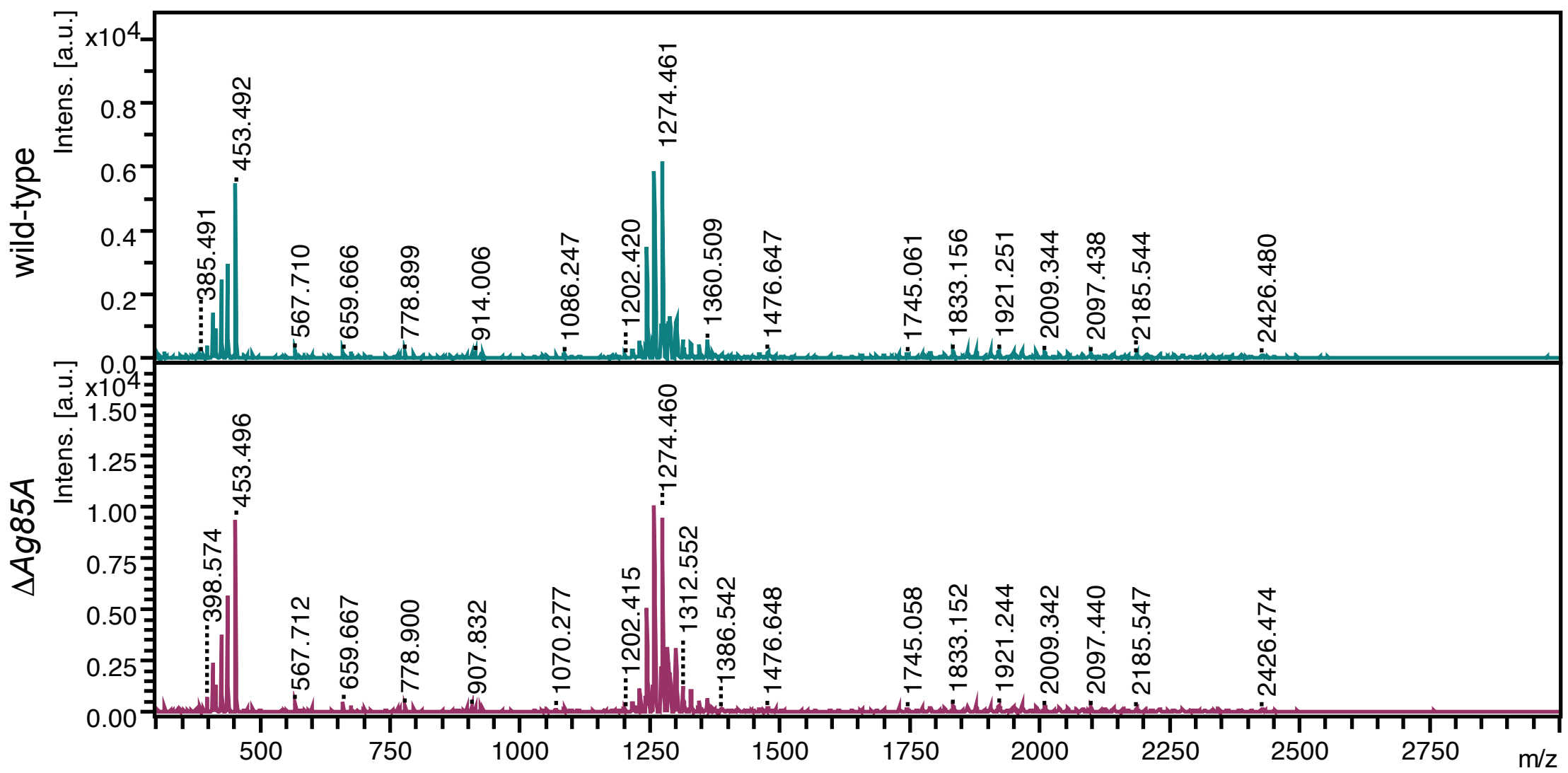

Supplementary Figure 4. Matrix-assisted laser desorption/ionization time-of-flight mass spectrometry (MALDI-TOF-MS) analysis of lipids extracted in Fig. 3D. The 1-butanol extract was analyzed by a Bruker UltrafleXtreme MALDI-TOF/TOF using 2,5-dihydroxybenzoic acid (DHB) as a matrix in positive-ion mode. An acceler-ating voltage of 20,000 V was applied. Peaks at m/z 1689 and 1717 correspond to the calculated masses of the sodium adducts of LOS-A.
